# Supplementary material for: The Superiority of TiO2 Supported on Nickel Foam over Ni-Doped TiO2 in the Photothermal Decomposition of Acetaldehyde
Source: Materials (Basel). 2023 Jul 26;16(15):5241. doi: 10.3390/ma16155241 (PMC10420295; doi:10.3390/ma16155241)
Supplement: Supplementary file 1 [file materials-16-05241-s001.zip › materials-2490280-supplementary.pdf]

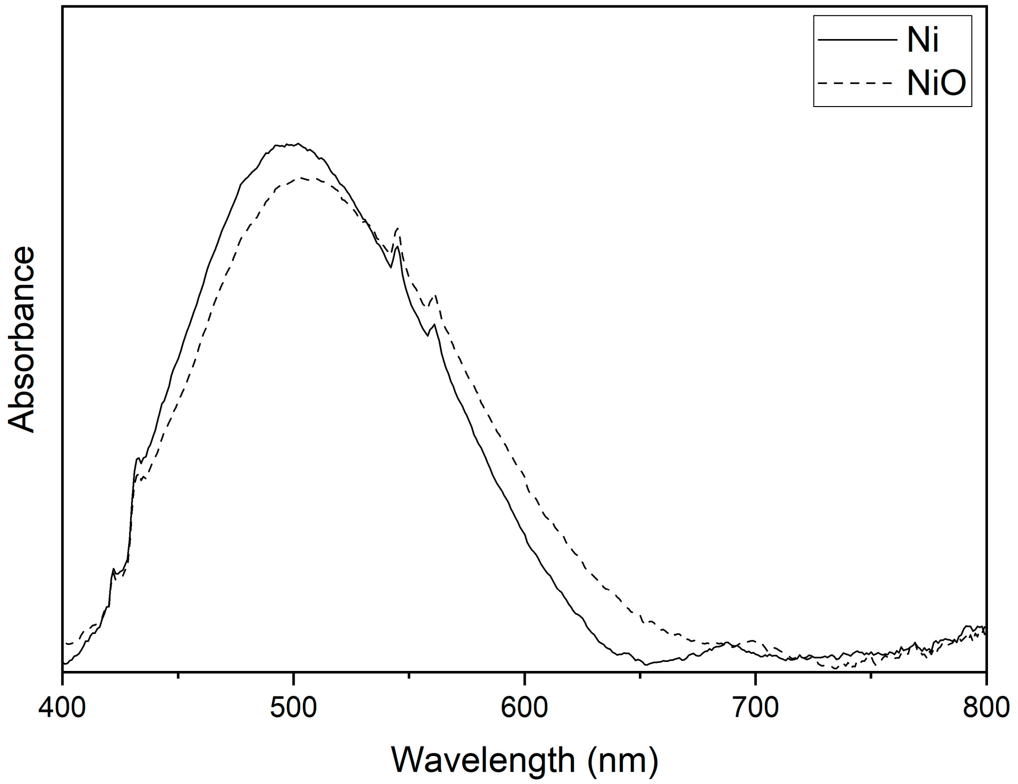

Figure. S1. UV-Vis spectra of TiO_2 doped with $AgNO_3$ and supported on nickel foam (Ni) and oxidised nickel foam (NiO) after irradiation for 10 min under UV LED light
